# Randomized clinical trial of medial unicompartmentel versus total knee arthroplasty for anteromedial tibio-femoral osteoarthritis. The study-protocol

**DOI:** 10.1186/s12891-019-2508-1

**Published:** 2019-03-20

**Authors:** Jacob Fyhring Mortensen, Lasse Enkebølle Rasmussen, Svend Erik Østgaard, Andreas Kappel, Frank Madsen, Henrik Morville Schrøder, Anders Odgaard

**Affiliations:** 1 Department of Orthopedic Surgery, Copenhagen University Hospital Herlev-Gentofte, Kildegårdsvej 28, DK-2900 Hellerup, Denmark; 2 Department of Orthopedic Surgery, Vejle Hospital, Kabbeltoft 25, DK-7100 Vejle, Denmark; 3 Department of Orthopedic Surgery, Aalborg University Hospital, Hobrovej 18-22, DK-9100 Aalborg, Denmark; 4 Department of Orthopedic Surgery, Århus University Hospital, Tage-Hansens Gade 2, DK-8000 Aarhus, Denmark; 5 Department of Orthopedic Surgery, Næstved Hospital, Ringstedgade 61, DK-4700 Næstved, Denmark; 6 Department of Orthopedic Surgery, Gentofte Hospital, Kildegårdsvej 28, DK-2900 Hellerup, Denmark

**Keywords:** Medial Unicondylar knee arthroplasty, Total knee arthroplasty, OKS, Anteromedial osteoarthritis, Knee replacement

## Abstract

**Background:**

In treatment of isolated medial unicondylar osteoarthritis of the knee, it is possible to choose between medial unicondylar knee arthroplasty (mUKA), or a total knee prosthesis (TKA). The demand for a blinded multicenter RCT with the comparison of mUKA and TKA has been increasing in recent years, to determine which prosthesis is better. Supporters of TKA suggest this treatment gives a more predictable and better result, whereas supporters of UKA suggest it is unnecessary to remove functional cartilage in other compartments. If the mUKA is worn or loosens, revision surgery will be relatively easy, whereas revision-surgery after a TKA can be more problematic.

**Methods:**

A double-blinded multicenter Randomized Clinical Trial setup is the aim of the study. 6 hospitals throughout all 5 municipal regions of Denmark will be participating in the study. 350 patients will be included prospectively. Follow-up will be with PROM-questionnaires and clinical controls up to 20 years.

**Discussion:**

Results will be assessed in terms of 1) PROM-questionnaires, 2) Clinical assessment of knee condition, 3) cost analysis. To avoid bias, all participants except the theatre-staff will be blinded.

**PROMs:**

OKS, KOOS, SF36, Forgotten Joint Score, EQ5D, UCLA activity scale, Copenhagen Knee ROM scale, and Anchor questions. Publications are planned at 2, 5 and 10 years after inclusion of the last patient. The development of variables over time will be analyzed by calculating the area under the curve (AUC) for the variable relative to the initial value, and comparisons of the between-group differences will be based on parametric statistics. In this study, we feel that we have designed a study that will address these concerns with a well-designed double-blinded multicentre RCT.

**Trial registration:**

ClinicalTrials.gov ID: NCT03396640.

Initial Release: 09/19/2017.

Date of enrolment of first participant: 10/11/17.

## Background

The causes of knee joint degenerative disease are multifactorial. It is known that trauma (resulting from meniscus, ligament or cartilage lesions), overload (obesity, malalignment), other disorders (e.g. rheumatoid arthritis) and genetic reasons all play a role in osteoarthritis (OA). Primary OA is knee degenerative disease with unknown aetiology [1]. The knee has three compartments, all of which can suffer from OA and there can thus be seen tri-, bi- or unicompartmental OA. The bicompartmental OA can occur in three combinations, where the combination of medial and patellofemoral involvement is the most frequent [2]. In most patients with knee unicompartmental OA, the arthritis is confined to the medial compartment [3], where it is most often anteromedial (AMOA).

**Fig. 1:**
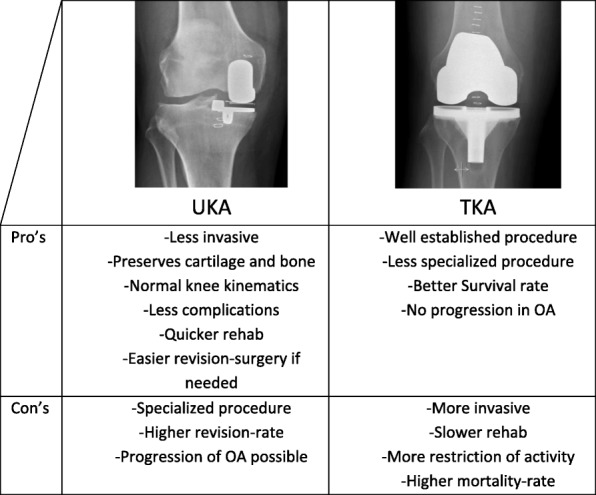
Pros and Cons of each Knee-replacement

AMOA can be treated with total knee arthroplasty (TKA) or medial unicompartmental knee arthroplasty (UKA) (Fig.1). In Denmark, the proportion of UKA has increased during the period 2013–2015 from 11% (2699/24574), to 16,8% (1344/7980) in 2016 [4]. The proportion may be expected to increase to above 30% in line with the proportion in the hospitals that are the most frequent users, if the general perception of the outcome remains positive. Despite the increasing usage, UKA is still controversial. According to a recent comparative meta-analysis of 374,934 arthroplasties, revisions of mUKA occur at an annual rate of 2.18-fold that of TKA [5]. In a survival analysis of a high-volume centre, the 9-year survival rate was 93% [6].

TKA-followers believe that the isolated AMOA is best treated with total knee arthroplasty (TKA) which is considered the gold standard, while UKA-followers believe that the TKA is an unnecessarily large intervention as it is more invasive and removes potentially good cartilage. UKA preserves bone and cartilage which makes revision surgery easier if necessary [7–10]. “In addition, mUKA has fewer complications, requires less rehabilitation, and may provide a better range of motion and superior function compared with total knee arthroplasty. Because it is associated with a higher risk of revision compared with that of total knee arthroplasty, unicompartmental knee arthroplasty remains a controversial procedure” [10]. A large register study from England and Wales [11] found that UKAs had worse implant survival than TKA, but less complications. They found worse UKA survival with revision (sub hazard ratio 2.12) and revision/reoperation (1.38) than TKAs at 8 years. In the same study, mortality was found significantly higher for TKA at all time points than for UKA (30 day: 0,23; 8 year: 0,85). Length of stay, complications, and rate of readmission were all higher for TKA than UKA [11].

When comparing UKA and TKA, findings are open to bias, especially comparisons of revision rates from national registries [12]. Murray et al. explain this as a selection bias due to UKAs tending to be implanted in younger, fitter and more active patients than TKA, while younger, fitter and more active patients tend to have higher revisions rates. Also they explain a measurement bias, as the decision to revise is influenced by the type of arthroplasty. Since UKA is generally easier to revise than TKA, and the outcomes of revision are considered to be better, the threshold for revision of UKA is lower than for TKA [12].

Over the coming years an increase in the number of knee replacements is expected [13]. Effective treatment with good clinical outcomes assessed in relation to the treatment costs will be an ever-increasing requirement for cost-effectiveness and maximizing usage of resources. There are no comparative studies on the cost and economic effectiveness between mUKA and TKA. While there are register studies without adequate scientific evidence, some still believe that register studies should rule the ultimate decision in the quality of implant types and brands, as well as their continued usage [14–16]. Data from register studies is being used more and more in the supply and sales processes. Some believe that the use of UKA implants should be reduced or stopped as a consequence of their poor performance in registry studies [16], yet some surgeons prefer the use of mUKA when possible, which is reflected in the increased rate of mUKA operations [4]. Murray et al. believe that these registries usually are biased against the UKA because of the way they are setup, which leads to selection-, reporting-, and measurement-bias [12, 17].

Limited evidence is available to clarify whether AMOA should be treated with a UKA or TKA. The aim of this study in a double-blinded randomized multicentre design is to compare the outcome of UKA and TKA for AMOA. Studies are currently on-going in the United Kingdom (TOPKAT) [3], Finland [18], and Sweden [19] to compare the two prosthesis. As suggested in the peer-review commentary on Kulshrestha et al., We await the results of a large multicentre randomized trial to provide information on whether specific patient populations can derive greater benefit from UKA’ [20]. Murray et al. also conclude that: ‘For a fair comparison of UKA and TKA, data from randomized controlled trials are required, and multiple outcome measures should be used which could include adverse events, patient reported outcome measures and cost-effectiveness’ [12].

In this study, we feel that we have designed a study that will address these concerns with a well-designed double-blinded multicentre RCT.

## Methods

The study has been designed as a superiority type, prospective, double-blinded, parallel-group, multicentre randomized clinical trial (RCT). Each participant is by lot [1:1] randomized to either UKA or TKA for the treatment of AMOA. This trial will comply with the CONSORT 2010 Statement [21] and the SPIRIT guidelines [22] and a Statistical Analysis Plan (SAP) [23] will be included.

### Participants

350 participants will be included consecutively in the study, from 6 participating hospitals covering all 5 regions nationally. Participating hospitals include Gentofte, Næstved, Vejle, Århus, Farsø and Frederikshavn hospitals.

**Inclusion/exclusion** criteria are listed in Table 1.

**Table 1:** Inclusion and Exclusion Criteria

Inclusion Criteria	Exclusion Criteria: Non-knee specific	Exclusion Criteria: Knee-specific
- AMOA severe enough to justify arthroplasty - Diagnosis ensured by standard x-ray with PA, lateral, and skyline - Bone-on-Bone medially - Lack of effect of conservative treatment	- Non-danish citizenship - Insuff. Danish capability - Under 18 years - Dementia - Severe Psych. Disorder - Alcohol og drug abuse - Disseminated malignancy - Severe systemic disease - Rheumatoid Arthritis - Employed at one of the participating departments	- Sagittal/coronal instability - Lateral and/or patellofemoral OA - Complex regional pain syndrome - Arthrofibrosis - Extension defect < 10 - Fleksion < 110 - Lateral subluxation or bone-on-bone visualized on skyline.

### Flow

**Fig. 2:**
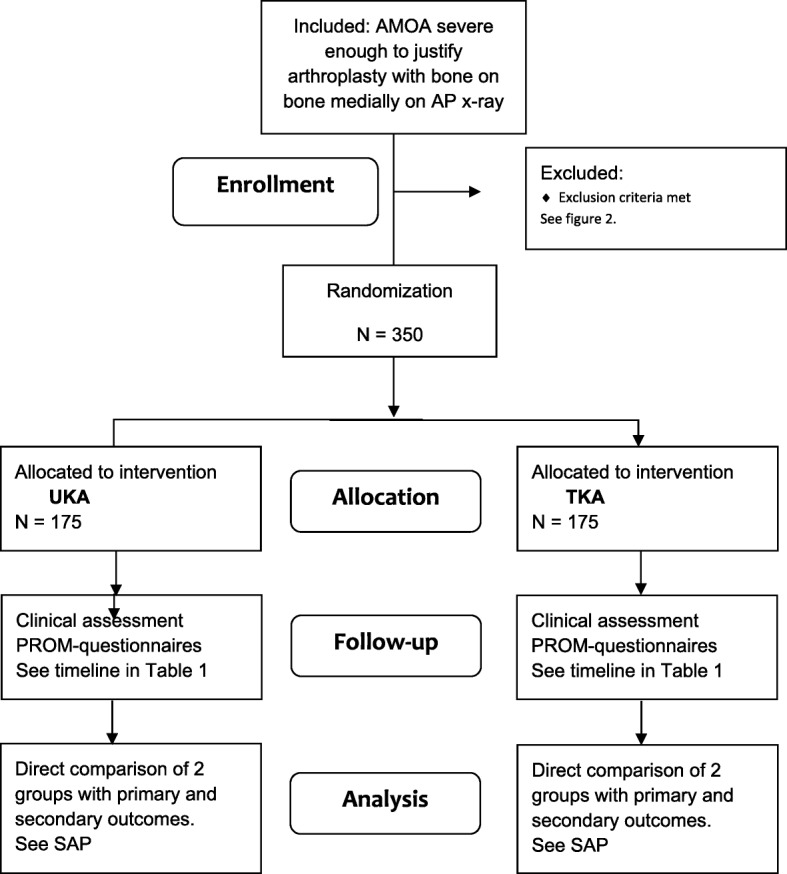
Consort Flow Diagram

Patients with isolated AMOA, who are referred to one of the participating departments, will be screened for eligibility by a knee-surgeon and will be offered inclusion in the study (see Table 1 and Fig. 2). The patient will then receive verbal and written information regarding the study, and will consider participation, after signing the consent form, which is given to the surgeon. Within 3 days the patient will be contacted by one of the investigators ensuring that the patient has read and understood all the received information, and they will be given the opportunity to ask questions. If they consent, prior to surgery they must answer baseline questionnaires and have baseline a surgical clinical evaluation. Pre- and postoperative follow-up is carried out according to Table 2.

**Table 2:** W = weeks, M = months, Y = years, PROM = Patient Reported Outcome Measures, P/E = Physical examination, Comp = complications, # = Unblinding of patient treatment

	Enrolment	Allocation	PROM	P/E	X-ray
Eligibility screening	**●**				
Informed consent	**●**				
Baseline variables			**●**	**●**	**●**
Operation		**●**			
2w				**●**	**●**
1 m			**●**		
2 m			**●**		
3 m			**●**		
4 m				**●**	
6 m			**●**		
9 m			**●**		
1y		**#**	**●**	**●**	**●**
18 m			**●**		
2y			**●**	**●**	**●**
3y + 4y			**●**		
5y			**●**		
6y + 8y			**●**		
10y			**●**	**●**	**●**
12y + 14y + 16y + 18y			**●**		
20y			**●**	**●**	**●**

### Intervention

Based on the randomization, the patient will have either: 1) Cementless medial Oxford partial knee, phase 3-alpha, or 2) Cemented TKA.

The TKA brand and model will be the individual surgeon’s preferred TKA. General anaesthesia or spinal analgesia will be used depending on the surgeon and anaesthetist preferences. The usage of a drain is also at the discretion of the surgeon. Local infiltration analgesics will be used. All patients will undergo surgery using a midline skin incision, and the length of UKA incisions will be as a TKA incision to ensure blinding postoperatively. After the skin incision, the joint-capsule will then be.

incised according to the standard operation technique of either prosthesis. All pre-, per-, and post-operative regimes of each department will be followed besides the regime described above. Surgeons from 7 centers with long experience of UKA will participate. The individual surgeon should have a personal proportion of at least 20% medial UKA of primary procedures for at least three months before study participation to have a homogenous group of surgeons in the trial, and to minimize the risk of revision [6].

### Primary outcome measure

**Oxford Knee Score (OKS)** is the primary outcome measure. OKS is a patient reported outcome measure (PROM) questionnaire with a scale range from 0 (severe arthritis) – 48 (satisfactory joint function) [24]. OKS consists of 12 simple questions and is easy to fill out for the patient. There are a low number of incorrectly completed forms [24, 25], and the form can be answered in a short time. OKS has been shown to be sensitive and OKS has been translated to and validated in multiple languages [26, 27].

### Secondary outcome measures

Patient reported outcome measures.

A compiled questionnaire containing a number of generic and disease-specific PROMs will be sent to participants to be answered pre-operatively and post-operatively at 1–2–3-6-9-12-18 months and at 2–3–4-5-6-8-10-12-14-16-18-20 years (see Table 2).

#### SF36

Evaluating individual patients’ generic health status This PROM has the potential to allow assessments of cost-effectiveness, and monitoring/comparing disease burden [28].

#### EQ5D + EQ5D vas

Standardized instrument for measuring generic health status, without and with a visual analogue scale, respectively [29].

#### Forgotten joint score

The patients ability to forget about a joint as a result of successful treatment [30, 31].

#### KOOS

The score is based on the patient’s own assessment and is an extension of WOMAC, which can also be calculated from the completed form [32, 33].

#### UCLA activity scale

Assessment of patient activity outcome evaluations of lower extremity joint reconstructions [34, 35].

#### Copenhagen knee ROM scale

Patient reported knee range of motion, picture based [36].

The Oxford Knee Score, KOOS and SF36 for quantification of knee function, lower extremity function, and psychosocial status. In addition to being well documented, the patient-reported outcome measures (PROMs) are based on self-assessment, allowing completion of forms by mail/email, which is a great advantage. WOMAC will be calculated from the KOOS. The Forgotten Joint Score is also relevant to use, as the patient-awareness of the artificial knee-joint or lack thereof, correlates to the success of the treatment. Some could argue that there could be a ‘ceiling effect’ using PROMs, but as seen with previous experience, almost no patients score maximum of points [37].

Knee score of KSS is not considered as relevant in specific measurements, and as an example, ROM in the clinical follow-ups will be of greater value. Unfortunately, OKS is not entirely specific, since it has been found that major hip or spine disease can affect the score [25]. However, all patients will be randomized, and confounders should be equally distributed between the two groups. HSS score and KSS have been shown to be influenced by demographic characteristics [38], and this may also be the case for OKS, KOOS and SF-36. This motivates the recording of the level of education and co-morbidity, such that randomization may be checked for this.

### Clinical outcome measures

Physical examinations will be performed at baseline and post-operatively at 2 weeks, 4 months, and 1, 2, 10 and 20 years. If a clinical control after 2 weeks cannot be performed, a telephone call will be performed instead.

#### Knee ROM

The number of degrees an examiner is able to move the knee joint through its full range of motion with no active effort from the patient (passive movement). Mobility is measured in degrees using a standard (30 cm) goniometer.

#### Knee effusion

Measured bilaterally as the circumference with the measuring band placed 1 cm above the base of the patella [39].

#### Implant survival

Whether the patient has undergone revision surgery of the arthroplasty or other additional surgery to the knee. The information will be retrieved for the National Patient Register (NPR) and from the Danish Knee Register (DKR).

#### Complications

Fracture, infection, loosening, radiographic radiolucent line, thromboembolism, other.

### Cost utility analysis

The hospital’s accounting department and product suppliers will provide unit costs, which will be combined with data from the Patient administrative system (PAS), data from the regional health insurance registry, and data from Statistics Denmark [40]. Over several years fees collected will be adjusted for inflation and presented in prices for the most recently observed year.

QALYs measured with EQ5D will provide endpoints of the CEA. Quality of life (QOL) will be calculated using the official Danish time trade off (TTO) from the baseline and with each follow up [41]. QALY is based on observed values QOL and calculated for a whole year by estimating the area under the curve (AUC) with the Trapezoidal rule.

Scored by methods described above, secondary endpoints are SF36 and OKS.

Estimation of univariate and multivariate cost difference will be calculated between the two treatments.

Due to an expectation of a non-normal distribution of cost and effect data, primary univariate analysis carried out will be as a non-parametric bootstrap with arithmetic mean values based on 2000 bootstrap samples [42]. A simple T-test will be the secondary univariate analysis [43].

Means of generalized linear method (GLM) that allow investigating the cost without requirements for data distribution will be used for the multivariate analysis of costs. Following covariates are included in the model: age, gender, diagnosis, preoperative costs, economically active/not, and pre-operative QOL.

Means of linear regression (OLS) will be used for multivariate analyses of the impact, with bootstrap sampling of 2000 samples. Following covariates in the model will be included: age, gender, diagnosis, economically active/not, and pre-operative QOL [42].

Cox regression will be used additionally at long-term follow-up.

Missing data in any analysis will only be replaced after current conventions for used measuring tools, using multiple imputations. Therefore all analysis can be carried out on complete sets. The estimated net benefit (NMB) uses the following formula: NMB = R_T_ △E - △*C*.

R_T_ will be the threshold ratio and will be set for 50,000 US dollars. If the NMB is > 0, the treatment is cost-effective. Data will be presented with figures of uncertainty about ICER, along with bootstrap samples of 2000, with paired observations of cost- and power difference on the cost-effectiveness axis.

On the y-axis, cost data will be allocated. On the x-axis, effect data. The four quadrants describe the relationship between the two investigated treatments.

In the northeast quadrant the UKA is more expensive, but superior. In the southeast quadrant, the UKA will dominate the TKA. In the southwest quadrant UKA is cheaper but poorer than TKA. In the northwest quadrant, the TKA is dominant, meaning the UKA is both more expensive and poorer. The probability of the UKA method will be described by data with an acceptability curve. A net benefit figure showing the result of the NMB analysis at different values of W (willingness to pay) will also be shown.

### Sample size

Based on previous studies [37], the difference between pre- and postoperative OKS has a standard deviation around 8.4. The minimally clinically important difference for OKS is 3. Setting the significance level (alpha) at 5% and the power (1-beta) at 90%, 170 patients are needed in each group resulting in a total study population of 340 patients. This number has been rounded up to 350 by the research-group.

## Randomization

Stratified, permuted block randomization with a 1:1 allocation ratio. Block sizes will be 4, 6, 8 and 10. Stratification will be two-dimensional, where hospital is one dimension and patient sex the other. The randomization will be done as shortly before surgery as possible.

The random allocation sequence is made digitally by the coordinating investigator, using a unique database www.procordo.dk.

Enrolment of participants occurs in the outpatient clinics of the participating orthopaedic departments, where all knee surgeons screen patients’ inclusion/exclusion criteria and offer them inclusion in the trial as appropriate. Prior to surgery the participant will be randomized on-line by the surgeon. The staff will be advised immediately after randomization, which procedure will be performed. If the participant is allocated to. the UKA group and during surgery shows to have widespread arthritis in the knee, the patient will receive a TKA, but will remain in the study and remain blinded for 1 year. Hence, analyses will be according to intention-to-treat principles.

### Blinding

**Fig. 3:**
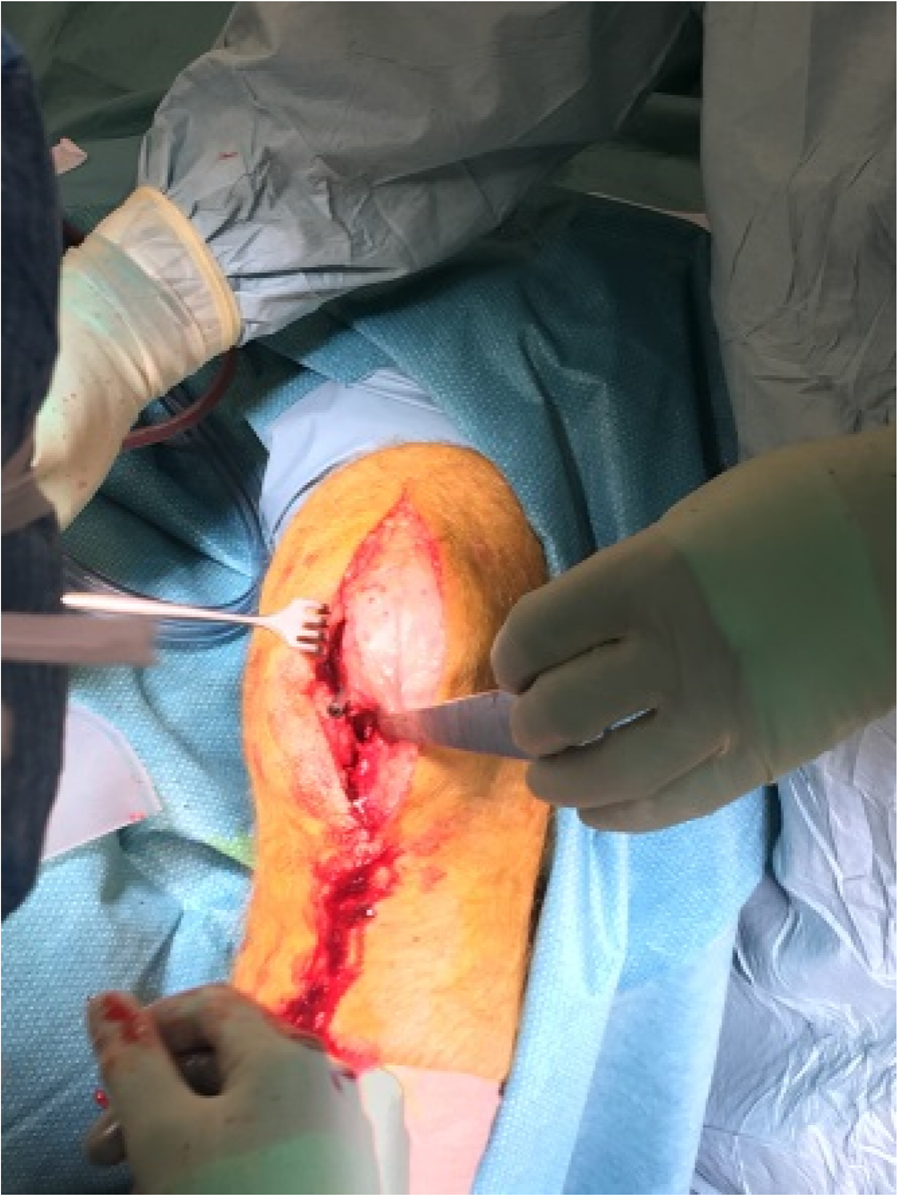
Midline incision of knee, with medial capsulotomy for mUKA

Randomization is blinded to everyone (patient, staff, GP, physiotherapists etc.) except theatre staff, during the first year following surgery. Blinding is secured by making a midline skin incision of the knee, regardless of randomization. If a UKA is performed, the skin is pulled medially, and the joint capsule is incised medially as the standard operation manual dictates (Fig. 3). After surgery, the patient will have numerous contacts with different types of medical staff. All medical staff involved is thoroughly instructed in the principles of blinding, and are themselves blinded to the operation notes, radiographs and discharge summaries. Post-operative control x-ray will be done at the 2-week clinical follow-up, or shortly before discharge. The radiographic wards are strictly instructed not to reveal the x-ray result to the participant. In case of accidental unblinding of staff, they are instructed not to share the information with the participant. Participants are asked at all contacts whether their randomization has been revealed, and if so, how.

### Statistical analysis plan

Changes between pre- and postoperative status will be treated with paired statistics, either parametric or nonparametric depending on the nature of the data. The development of variables over time, e.g. Oxford Knee Score, will be analysed by calculating the area under the curve (AUC) for the variable relative to the initial value, and comparisons of the between-group differences will be based on parametric statistics.

Statistical comparison of proportions in the two treatment groups will be conducted with chi-squared –test or Fisher’s exact test. The method is used each time a defined serious adverse event (SAE) is recorded. Complications, in contrast to SAE, are divided into knee- and non-knee related groups. Knee related complications include revision, re-operation, loosening, deep infection, superficial infection, unexplained pain, pain, and lack of improvement in OKS after 6 months compared to pre-operatively. Non-knee related complications include DVT with following thromboembolism, anaesthesiology complication, and death. Out of these complications, the study group defined Death, Infection, and lack of improvement in OKS at 6 months compared to pre-operatively as an SAE.

All analyses will be based on an ‘Intention-to-treat’ basis. Statistical significance will be judged with 95% confidence intervals presented.

Analyses are planned early at 2 years after the last participant is included, medium at 5 years, and late at 10 years follow-up of last participant.

### Interim analysis

The Institutional review board required that a Data Review Board be set in places, which are neutral to the study. Running, the Board will be informed about any SAE and the board will be gathered after inclusion of 33 and 66% of the patients. A possible difference between the groups will be tested for statistical significance by contingency analysis (2 × 2 table, chi square test) where *p* < 0,025 will be regarded significant. The reason for choosing this low *p*-value is that this study group regard this study to be of high priority, and to protect patients in the future, that this study only be closed down when an absolute difference between the interventions can be shown.

Also, at each event of SAE, previous cases of SAE’s will be analysed, and it will be determined whether one of the groups has a significant excess risk of complications, as written above. If it has, the trial will be stopped, and the results published.

### Timeline and ethics

Recruitment of patients started September 2017. Based on a patient acceptance ratio of 50% and the current number of partial knees performed in the participating centres, inclusion is expected to last between one and two years as a conservative estimate.

The trial protocol has been approved by the Regional Ethics Committee of the Capital Region of Denmark. It has been amended once, latest on the 18/03/2018.

## Discussion

The investigating group has designed this RCT to address international debates whether Oxford medial UKA is better than TKA for the treatment of AMOA, as predictions of future demands and usage of medial UKA are expected to increase. Other similar studies are on-going, but unfortunately they are lacking in a study-design that will properly determine superiority of these two interventions. The TOPKAT-study in the UK [3] was an unblinded study, which unfortunately will result in too much bias and uneven spreading of confounders. The Finnish FUNCTION-trial [18], is well designed, but this study group has the opinion that a sample-size group of 140 patients is too low, and will not have sufficient power in their study, same as the Swedish trial [19] with only 80 patients, although their primary outcome is on muscle mass change results.

The outcome of this trial will provide high-level evidence as to whether a TKA or UKA is beneficial in both the long and the short term. As the trial will be double-blinded and performed as a multi-centre study, potential biases are reduced. Results with primary focus: 1) patient-reported outcomes, 2) clinical results including prosthetic survival, and 3) costs.

If the trial results in a high drop-out, a drop-out analysis will be performed.

Results will be submitted to peer-reviewed international journals for publication in accordance with the CONSORT guidelines for reporting of clinical trials.

## Abbreviations

AMOA: AnteroMedial OsteoArthritis
AUC: Area Under the Curve
DKR: Danish Knee Register
EQ5D: Euroqol-5 levels of severity
EQ5D-VAS: Same as above, with Visual Analogue Scale
FJS: Forgotten Joint Score
GLM: Generalized Linear Method
ICER: Incremental Cost-Effectiveness Ratio
KOOS: Knee Injury and Osteoarthritis Outcome Score
KSS: Knee Society Score
mUKA: Medial Unicompartmental Knee Arthroplasty
NMB: Net Monetary Benefit
NPR: National Patient Register
OA: Osteoarthritis
OKS: Oxford Knee Score
PAS: Patient administrative system
PROM: Patient Reported Outcome Measure
QALY: Quality Adjusted Life Year
QOL: Quality Of Life
RCT: Randomized Clinical Trial
ROM: Range Of Motion
SAE: Serious Adverse Events
SAP: Statistical Analysis Plan
SF36: Short Form 36
TKA: Total Knee Arthroplasty
TTO: Time Trade Off
